# Real-World Effectiveness and Safety of Photoimmunotherapy for Head and Neck Cancer: A Multicenter Retrospective Study

**DOI:** 10.3390/cancers17162671

**Published:** 2025-08-16

**Authors:** Isaku Okamoto, On Hasegawa, Yukiomi Kushihashi, Tatsuo Masubuchi, Kunihiko Tokashiki, Kiyoaki Tsukahara

**Affiliations:** 1 Department of Otorhinolaryngology, Head and Neck Surgery, Tokyo Medical University, 6-7-1 Nishishinjuku, Shinjuku-ku, Tokyo 160-0023, Japan; m03075kt@tokyo-med.ac.jp (K.T.); tsuka@tokyo-med.ac.jp (K.T.); 2 Department of Oral and Maxillofacial Surgery, Tokyo Medical University, 6-7-1 Nishishinjuku, Shinjuku-ku, Tokyo 160-0023, Japan; on-h@tokyo-med.ac.jp; 3 Head and Neck Oncology Center, International University of Health and Welfare, Mita Hospital, 1-4-3 Mita, Minato-ku, Tokyo 108-8329, Japan; ykushihashi32@gmail.com (Y.K.); tmasubuchi@chibanishi-hp.or.jp (T.M.)

**Keywords:** photoimmunotherapy, head and neck cancer, cetuximab sarotalocan sodium, unresectable, advanced cancer, local recurrence, time to treatment failure, real-world data

## Abstract

Background/Objectives: Photoimmunotherapy for head and neck cancer (HN-PIT) is an emerging treatment for unresectable locally advanced or recurrent head and neck cancer. However, real-world data (RWD) are limited. This study examined the safety and effectiveness of HN-PIT. Methods: This multicenter, retrospective cohort study included 40 patients with unresectable locally advanced or recurrent head and neck cancers who underwent HN-PIT from January 2021 to August 2024. The primary endpoint was time to treatment failure (TTF). Secondary endpoints included the objective response rate (ORR), overall survival (OS), progression-free survival (PFS), and adverse events (AEs). Results: The median TTF and 1-year treatment failure rate were 6.0 months and 23.2%, respectively. Moreover, the ORR, disease control rate, median OS, and median PFS were 75.0% (95% confidence interval [CI]: 60.0–86.0%), 95.0% (95% CI: 83.5–99.0%), 26.9 months, and 6.2 months, respectively. The incidence of grade ≥3 AEs was 17.5% (95% CI: 7.1–29.1%). Pain was the most common AE, occurring in 37 patients (92.5%), with grade III pain reported in 5 (12.5%). Mucositis occurred in 32 patients (80.0%), with grade III mucositis reported in 3 (7.5%). Hemorrhages occurred in 31 patients (77.5%), with no grade ≥III hemorrhages reported. Two patients experienced sepsis (5.0%; grades IV and V). Seventeen patients (42.5%) had laryngeal edema, with grade IV edema reported in four (10.0%). Conclusions: Our RWD shows that HN-PIT is effective with an acceptable safety profile. TTF may serve as an endpoint reflecting this treatment’s characteristics. This study provides important basic data for the development of future treatment strategies.

## Simple Summary

Photoimmunotherapy is a treatment strategy for unresectable head and neck cancer that combines the use of a light-sensitive drug and laser light. This study analyzed real-world treatment outcomes from multiple hospitals in Japan. Among 40 study patients, most experienced pain and mucositis; however, the treatment showed good disease control. The median time to treatment failure (TTF), median overall survival, and objective response rate were 6.0 months, 26.9 months, and 75%, respectively. Severe adverse events, including sepsis and laryngeal edema, were observed in some patients, while photoimmunotherapy for head and neck cancer (HN-PIT) had an acceptable safety profile. This study provides the largest real-world clinical dataset on HN-PIT, to date, and demonstrates TTF as a useful endpoint.

## 1. Introduction

Recent global cancer statistics indicate that head and neck cancer constitutes more than 900,000 new cases annually, with many of these being unresectable at diagnosis or post-recurrence [1,2]. Recurrent/metastatic head and neck squamous cell carcinoma (R/M HNSCC) carries a poor prognosis due to aggressive progression and therapeutic resistance [3]. For patients with R/M HNSCC, the main treatment aim is to prolong survival while enhancing quality of life (QOL). The head and neck contain organs that are essential for speech, swallowing, and breathing—functions that have a substantial impact on a patient’s QOL. Effective local control of head and neck cancer is believed to maintain patients’ QOL and prolong their survival. Photoimmunotherapy (PIT) was developed to support such local control.

PIT exerts its therapeutic effects through highly selective targeting of tumor cells while sparing surrounding healthy tissue [4,5]. This selectivity originates from the antibody component that specifically binds to molecules overexpressed on tumor cells. PIT induces immunogenic cell death, thereby stimulating antitumor immunity through dendritic cell activation and T-cell priming [6]. These effects help modulate the tumor microenvironment by enhancing the infiltration of immune cells, thereby promoting systemic antitumor responses beyond the illuminated area [7]. PIT is a cancer treatment method that has recently gained traction. It comprises the use of drugs containing photosensitive substances and laser light of a specific wavelength. These drugs are a conjugate of IR700, a photosensitive substance, and antibodies that bind to molecules specifically expressed in the treatment targets [4]. IR700 has a high absorption rate for red light with a wavelength of 690 nm. When red light is illuminated on the antibody–IR700 conjugate bound to the target molecule, the shape of the conjugate changes, inducing physical stress on the cell membrane. This increases transmembrane water flow, causing extracellular fluid to enter the cell, thereby resulting in swelling-induced cell rupture [5]. Such cell rupture leads to cancer cell necrosis [8].

The RM-1929-101 phase I/IIa trial conducted in the US (2015) was the first clinical evaluation of PIT for head and neck cancer (HN-PIT), establishing an optimal dose of 640 mg/m^2^ [9]. Moreover, a complete response (CR) rate, partial response (PR) rate, and unconfirmed objective response rate (ORR) of 13.3%, 30.0%, and 43.3%, respectively, were revealed in part 2 of the trial. The disease control rate (DCR), including that of patients with stable disease (SD), was 80.0%. In 2018, a phase I trial involving three Japanese participants was conducted to confirm the safety of PIT in this patient cohort [10]. Based on the trial’s results, PIT was included in the Sakigake Fast Track Review System for unresectable locally advanced or locally recurrent head and neck cancer (LA/LR-HNC) in Japan and received insurance coverage in 2021. HN-PIT has received conditional early approval, and phase III trials are currently under way. As of 2025, 4 years have passed since insurance approval in Japan, and real-world data (RWD) have been reported [11,12,13,14]. However, these reports are limited to data from single institutions, and no large-scale RWD on HN-PIT are available. Therefore, reports of larger numbers of HN-PIT cases are warranted. The aim of this study was to clarify the real-world outcomes of HN-PIT through a multicenter retrospective cohort, as previous reports were limited to single institutions or small cohorts.

## 2. Materials and Methods

### 2.1. Study Design

Patients’ medical records were analyzed in this multicenter, retrospective, observational study. The study was reported in line with the Strengthening the Reporting of Observational studies in Epidemiology statement [15] as well as tenets of the Declaration of Helsinki. As this was a multicenter study, it was conducted with approval from the ethics review board of each research institution to which the investigator belonged.

### 2.2. Patients

From 1 January 2021 to 31 August 2024, we enrolled patients aged 20 to 100 years who underwent HN-PIT for unresectable LA/LR-HNC at three departments: the Department of Otorhinolaryngology–Head and Neck Surgery and the Department of Oral and Maxillofacial Surgery at the Tokyo Medical University, and the Head and Neck Oncology Center at the International University of Health and Welfare, Mita Hospital. Patients were excluded if they had resectable disease or declined to participate.

### 2.3. Outcomes and Assessments

The primary endpoint was time to treatment failure (TTF), defined as the interval from HN-PIT initiation to discontinuation for any reason (disease progression, adverse events (AEs), or death). Secondary endpoints included ORR, overall survival (OS), progression-free survival (PFS), and AE incidence. OS was measured from the initiation of HN-PIT to death from any cause. PFS was defined as the time to either disease progression or death. ORR was based on the best overall response (BOR) per Response Evaluation Criteria in Solid Tumors (RECIST) version 1.1 [16]. The BOR assessment window was defined as the period from HN-PIT initiation to documented disease progression or the last imaging follow-up, whichever occurred first. Tumor staging followed the Union for International Cancer Control criteria, 8th edition [17]. AEs were categorized, following Common Terminology Criteria for Adverse Events version 5.0, into grades I–V, reflecting increasing severity from mild to death-related events [18]. Patients were monitored with imaging every 2–3 months, AEs were followed up until at least 3 months after treatment discontinuation. Missing data were addressed via complete-case analysis.

### 2.4. Photoimmunotherapy for Head and Neck Cancer

Treatment consisted of an intravenous infusion of cetuximab sarotalocan sodium at a dose of 640 mg/m^2^ over a period of at least 2 h. This was followed, 20–28 h later, by laser irradiation directed at the tumor site. Cetuximab sarotalocan sodium is a conjugate composed of cetuximab—an immunoglobulin G1 chimeric monoclonal antibody that targets human epidermal growth factor receptor (EGFR)—and IR700, a photosensitive dye. Owing to the light-reactive nature of IR700, the compound loses stability when exposed to illumination. Therefore, the illuminance must be limited to ≤120 lx during medication preparation. The infusion bag must also be enclosed with a special light-shielding cover.

Next, red laser light (690 nm) was delivered using the BioBlade laser system (Rakuten Medical KK, Tokyo, Japan). The laser system includes photodynamic therapy semiconductor lasers (BioBlade laser and BioBlade laser WR) and probes (including a frontal diffuser and side-fire diffuser for superficial illumination, as well as a cylindrical diffuser for tissue illumination). A diffuser guide tube was used as an auxiliary device for surface illumination, while a needle catheter was utilized for intra-tissue illumination. The illumination area was set with a safety margin of approximately 5–10 mm, depending on the target lesion location and size. Furthermore, the diffuser type was selected according to the illumination area, and surface illumination and/or internal tissue illumination were used. All procedures were performed under general anesthesia in an operating room.

**Figure 1:**
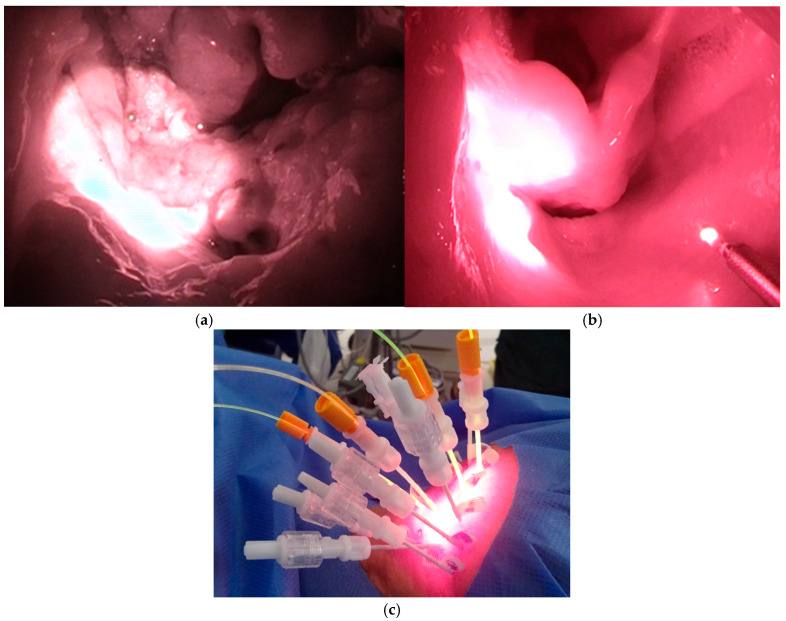
Photoimmunotherapy for head and neck cancer in clinical practice. (**a**) Frontal, (**b**) side-fire, and (**c**) cylindrical diffusers.

Surface illumination laser light was used for superficial lesions. Based on the laser equipment and output port employed, the laser light was illuminated in a circular pattern from the front or side, within a diameter ranging from 7 to 38 mm (Figure 1a,b). The illumination distance was approximately 1.7 times the spot diameter, whereas the target lesion was illuminated perpendicularly. Specifically, the illumination intensity (laser output density) was fixed at 150 mW/cm^2^, laser illumination time per session was 5 min 33 s, and light dose was 50 J/cm^2^. Illumination was performed in multiple sessions according to the required illumination area and working space. The cylindrical diffuser, which emits laser light in a cylindrical shape (Figure 1c), was inserted into the needle catheter after tissue puncture. This diffuser had a fluence rate of 400 mW/cm, and the laser illumination time per session was 4 min 10 s with a light dose of 100 J/cm. Needle catheters were available in lengths of 50, 70, and 100 mm. The appropriate length was selected based on the target lesion location and the approach to be used. Additionally, the required number of punctures was performed considering the cylindrical illumination area of each needle. Depending on the illuminated lesion condition, HN-PIT can be repeated for up to four cycles, a regimen deemed safe in a previous clinical trial [9], with at least a 4-week interval between cycles. Even if PD is observed post-HN-PIT, the treatment can be repeated if additional local control is needed and the lesion remains accessible to the laser.

### 2.5. Statistical Analysis

The TTF, PFS, and OS were analyzed using the Kaplan–Meier method. Statistical analyses were performed using EZR (version 1.68) [19].

## 3. Results

### 3.1. Patient Characteristics

A total of forty patients underwent HN-PIT during the study period. Table 1 presents the patients’ characteristics. The patients’ median age was 70 years (range: 36–87). Overall, 67.5% (27 individuals) were male. The oral cavity (17 patients, 42.5%) was the most common primary tumor site, followed by the oropharynx (7 patients, 17.5%) and nasopharynx (5 patients, 12.5%). Eighty cycles of illumination were performed, averaging two cycles per patient. The oropharynx (24 sites) was the most common target site for laser illumination, followed by the oral cavity (16 sites). All subsites of the illuminated areas were counted as the illuminated sites overlapped owing to lesion progression. Histological analysis revealed that 38 patients (95%) had squamous cell carcinoma, whereas the remaining two with non-squamous cell carcinoma were confirmed as EGFR-positive. Regarding treatment history, 31 (77.5%) and 38 (95.0%) patients had histories of surgery and radiotherapy, respectively. The primary site and target lesion were located in the oral cavity in the two patients without a history of radiotherapy.

### 3.2. Effectiveness

**Figure 2:**
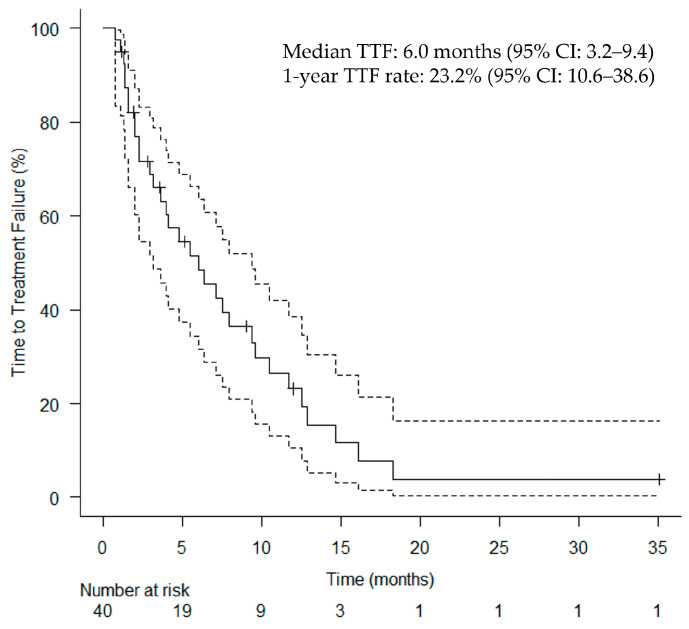
Kaplan–Meier curves of TTF. Vertical lines show censored events. CI, confidence interval; TTF, time to treatment failure.

**Figure 3:**
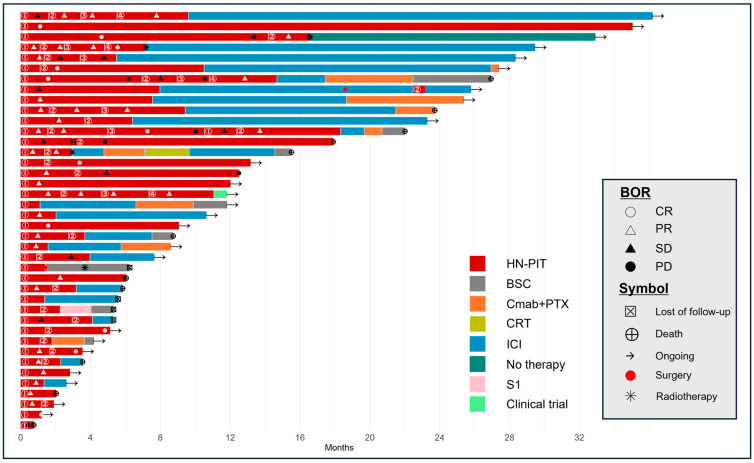
Swimmer plot of the clinical course of patients who underwent HN-PIT. Time to response and duration of survival (red portion of bar graph). Each bar represents a single patient, with the length of the bar corresponding to overall survival. HN-PIT, photoimmunotherapy for head and neck cancer; BOR, best overall response; BSC, best supportive care; Cmab, cetuximab; CR, complete response; CRT, chemoradiotherapy; ICI, immune checkpoint inhibitor; PD, progressive disease; PR, partial response; PTX, paclitaxel; SD, stable disease; S1, tegafur–gimeracil–oteracil.

**Figure 4:**
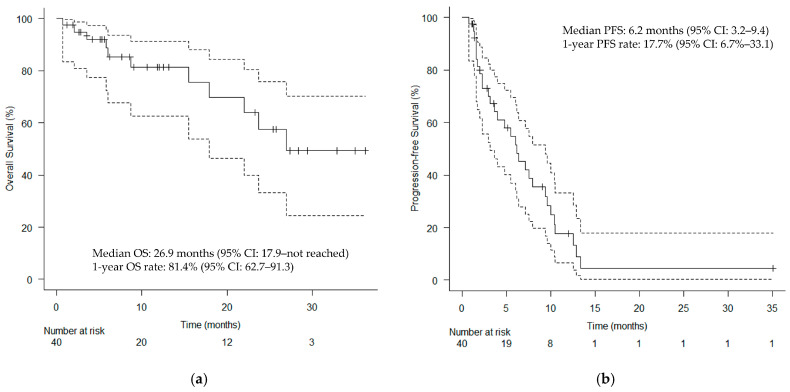
Kaplan–Meier curves. (**a**) OS and (**b**) PFS. Vertical lines show censored events. CI, confidence interval; OS, overall survival; PFS, progression-free survival.

The median follow-up duration was 9.9 months. The median TTF for the primary endpoint was 6.0 (95% confidence interval [CI]: 3.2–9.4) months (Figure 2). Figure 3 shows the clinical course of all patients who underwent HN-PIT, including sequential treatment, as a swimmer plot. The median OS and PFS were 26.9 months (95% CI: 17.9–not reached; Figure 4a) and 6.2 months (95% CI: 3.2–9.4; Figure 4b), respectively. Table 2 presents the BOR. The ORR and DCR were 75.0% (95% CI: 60.0–86.0%) and 95.0% (95% CI: 83.5–99.0%), respectively.

### 3.3. Safety

Table 3 presents all AEs. Pain was the most common AE, occurring in 37 patients (92.5%), with grade III pain reported in 5 (12.5%). Mucositis occurred in 32 patients (80.0%), with grade III mucositis observed in 3 (7.5%). Hemorrhages were observed in 31 patients (77.5%); however, no cases of grade ≥III hemorrhages were reported. The incidence of grade ≥III AEs was 17.5% (95% CI: 7.1–29.1%). Sepsis was observed in two patients (5.0%; grades IV and V). Seventeen patients (42.5%) had laryngeal edema, including four (10.0%) cases of grade IV edema.

## 4. Discussion

This multicenter series represents the largest RWD analysis of HN-PIT, to date. This represents the largest real-world study, to date, in terms of patients and treatment cycles. Regarding its effectiveness, the median TTF, median OS, median PFS, ORR, and DCR were 6.0 months, 26.9 months, 6.2 months, 75%, and 95%, respectively. All of these were favorable outcomes. The safety level was also within an acceptable range.

In the phase 1/2 RM-1929-101 trial [9], the unconfirmed ORR and DCR were 43.3% and 80.0%, respectively. Although these rates were more favorable in our study, we evaluated the ORR using the BOR. HN-PIT should be regarded as a combination of systemic therapy and laser illumination when evaluating effectiveness, rather than systemic therapy alone. If the effectiveness is evaluated using the method designed for systemic therapy, the results may differ from the actual therapeutic effect. Laser treatment is considered a type of surgery as many lesions temporarily shrink post-treatment. This raises the question of whether the ORR is an appropriate measure of the effectiveness of HN-PIT. As HN-PIT usually enables retreatment following PD, PFS is considered an inappropriate measure of its therapeutic effectiveness in clinical practice. Therefore, TTF was selected as the primary endpoint to evaluate the effectiveness of HN-PIT. Considering the specificity of HN-PIT, the period during which disease control is possible through local treatment may serve as a more appropriate measure of its effectiveness. Retreatment with HN-PIT remains a viable option in cases in which progression is limited to the local region. As shown in the swimmer plot in Figure 3, we determined that the most appropriate measure of effectiveness was the period during which local control was maintained with HN-PIT. The median PFS was 6.2 months in this study, which was slightly longer than the TTF of 6.0 months. This varied result may have resulted from the inclusion of patients who were switched to immune checkpoint inhibitor (ICI) treatment before PD in anticipation of the effects of immune activation induced by PIT. Preclinical experiments demonstrated that PIT, when combined with programmed cell death protein 1 (PD-1) inhibition, amplified antitumor effects by initiating tumor cell death, which in turn activated dendritic cells. This process led to cytokine production, T-cell stimulation, priming of antigen-specific immunity, and sustained memory responses [6,7]. Therefore, the present study included patients for whom curative treatment with PIT was not expected, and who were switched to ICI therapy before PD, in anticipation of the effects of immune activation on the tumor immune environment by PIT. This is the first study to employ TTF as an effectiveness measure for HN-PIT, and its results may serve as a reference for future clinical research on HN-PIT.

Although case reports on the effectiveness and safety of HN-PIT have been sporadically published [20,21,22,23,24,25,26,27,28], the two RWD studies involved <20 patients [11,14]. In the RM-1929-101 trial [9], the median OS and PFS were 9.30 (95% CI: 5.16–16.92) and 5.2 (95% CI: 2.10–5.52) months, respectively. However, our study revealed a median OS and PFS of 26.9 and 6.2 months, respectively. The favorable OS may be attributed to treatment administration at an earlier stage of local recurrence than is typically feasible in a clinical trial, as well as the effectiveness of the subsequent treatment administered post-HN-PIT in this study. As shown in the swimmer plot in Figure 3, PD-1 inhibitors were frequently selected as the next line of treatment, including for patients who exhibited PD despite undergoing PIT. HN-PIT-induced alterations in the tumor immune environment might have enhanced the effects of subsequent PD-1 inhibitor administration. A recent report indicated that pembrolizumab yields favorable outcomes after HN-PIT [21].

HN-PIT serves as one of the therapeutic strategies for patients with unresectable LA/LR-HNC, in addition to systemic treatments. According to the National Comprehensive Cancer Network guidelines [29], PD-1 inhibitors—specifically nivolumab and pembrolizumab—are recommended as first-line systemic agents in this setting. The CheckMate 141 trial [30] demonstrated the clinical benefit of nivolumab, with a reported median OS of 7.5 months (95% CI: 5.5–9.1) and median PFS of 2.0 months (95% CI: 1.9–2.1). Meanwhile, the KEYNOTE-048 trial [31], conducted to evaluate pembrolizumab monotherapy in patients with a combined positive score ≥1, showed improved outcomes, with a median OS of 12.3 months (95% CI: 10.8–14.9) and a median PFS of 3.2 months (95% CI: 2.2–3.4). Given that systemic therapy is a non-curative treatment for HN-PIT, a complete comparison cannot be made with our study. However, considering the survival rate with systemic therapy and the fact that tumor cell death induced by HN-PIT activates local and peripheral T-cell responses, HN-PIT administration followed by PD-1 inhibitors after treatment failure may be the best combination at this point.

Rakuten Medical KK is currently collecting surveillance data on post-marketing safety and has released an interim report on 157 patients as of September 2025. Common AEs included pain at the application site (53.5%), laryngeal edema (21.0%), dysphagia (14.0%), and facial edema (12.1%). In this study, 37 patients (92.5%) experienced pain, and 32 (80.0%) had mucositis, accounting for a considerable proportion of AEs. Pain management is extremely important as it affects patients’ QOL. Notably, our strategy is grounded in the concept of multimodal analgesia [32]. Laryngeal edema is another AE that requires attention. It occurred in 17 patients (42.5%) in our study, 4 of whom had grade IV edema. Notably, it occurred even in cases in which the direct target of PIT was not the larynx. Laryngeal edema can cause airway obstruction, which is a life-threatening AE. Therefore, measures must be in place for airway management via tracheostomy during HN-PIT. Prophylactic tracheotomy was not initially considered, except to treat the oropharynx and larynx. However, two patients required emergency tracheotomy owing to laryngeal edema-induced airway obstruction. Prophylactic airway management was subsequently implemented for all patients, except for those with a pre-existing permanent tracheostoma or nasopharyngeal cancer. Specifically, prophylactic tracheotomy or airway management via endotracheal intubation was performed in the intensive care unit. The most severe AE in this study was severe infection; one case each of grade IV and V sepsis was observed. Lemierre’s syndrome, which is another AE observed in this study, is triggered by infection. The patient with grade V sepsis originally had grade I mucositis but experienced fatal septic shock. This patient’s general condition changed rapidly during hospitalization on day 23 post-HN-PIT, indicating the necessity to carefully manage infection following PIT. The average length of hospital stay post-HN-PIT is approximately 2 weeks. Even if a patient with mucositis is discharged in good general health, their health may suddenly deteriorate at home owing to the rapid spread of the infection. Therefore, the patient should be carefully observed for approximately 1–2 months until resolution of the mucositis.

This study has certain limitations that should be considered. First, the definition of unresectable head and neck cancer is unclear regarding HN-PIT indications [33]. The factors contributing to a status of “unresectability” vary greatly among patients, surgeons, and institutions. Moreover, the treatment phase during which HN-PIT is administered is a major factor. Whether the treatment was administered after local recurrence post-local therapy or after PD post-systemic therapy may affect OS. As HN-PIT can be administered for up to four cycles, its treatment schedule requires careful consideration. Various opinions exist on this point. For example, some clinicians believe that only one cycle should be performed as curative treatment, with additional cycles to be considered if recurrence is confirmed. Long-term CRs were observed after a single cycle of HN-PIT in our analysis. Another approach involves the administration of regular treatments (every 4 weeks) for residual tumors, similar to the strategy used in systemic therapy. Furthermore, a single cycle of HN-PIT may be followed by an early transition to PD-1 inhibitor treatment to potentially elicit an immune response. In clinical settings, this decision is made according to each patient’s specific condition. Therefore, prospective studies on patient treatment courses and objectives are desirable.

Although QOL is an important consideration in head and neck cancer treatment, this study did not include specific QOL assessments owing to its retrospective design. As noted, pain (92.5%) and mucositis (80.0%) were commonly observed and likely had considerable short-term impacts on patients’ QOL post-treatment. This represents a limitation of our study. Future prospective studies should incorporate objective QOL assessments to better evaluate the impact of HN-PIT from the patient’s perspective. Furthermore, the inclusion of patients with tumors located in various anatomical sites and with diverse treatment histories may have introduced heterogeneity and potential confounding factors into the results. This is another study limitation. To evaluate potential immune synergy, a phase III trial (NCT06699212) is under way to investigate pembrolizumab in combination with HN-PIT as first-line therapy for recurrent head and neck squamous cell carcinoma. The results of that study are expected to provide important clinical insights.

Physicians treating patients with head and neck cancer are primarily focused on obtaining the best treatment outcomes without compromising QOL [11]. In this study, the median TTF for HN-PIT was 6.0 months, which is an important endpoint. The treatment algorithm for head and neck cancer prioritizes local treatment, followed by systemic therapy if local treatment is inadequate (owing to unresectable lesions or distant metastases), with a final transition to best supportive care. The incorporation of PIT into the treatment strategy for head and neck cancer may extend survival time, potentially reflecting the TTF observed with HN-PIT. HN-PIT may also have a positive effect on subsequent systemic therapy. Ultimately, an extension of OS in patients with head and neck cancer is anticipated. A direct comparison of the effectiveness of conventional PD-1 inhibitors and PD-1 inhibitors post-HN-PIT may yield valuable data, and we are planning further studies to explore this topic. However, a comparison of HN-PIT with systemic therapy to determine which is better is not meaningful. The critical focus should be on the sequential use of all treatments and drugs within the treatment algorithm for head and neck cancer.

## 5. Conclusions

The effectiveness and safety of HN-PIT were evaluated in 40 patients (80 cycles in total). Regarding its effectiveness, the median TTF, median OS, median PFS, ORR, and DCR were 6.0 months, 26.9 months, 6.2 months, 75%, and 95%, respectively. TTF proved to be a useful endpoint to assess the effectiveness of HN-PIT. Although safety was within acceptable levels, grade V AEs were observed, highlighting the need for careful follow-up post-HN-PIT.

## Figures and Tables

**Table 1:** Patients’ characteristics.

Characteristics	All Patients (*n* = 40)	
	Number	%
Age, years		
Mean	67	
Median	70	
Range	36–87	
Sex		
Male	27	67.5
Female	13	32.5
ECOG Performance Status		
0	32	80.0
1	7	17.5
2	1	2.5
Primary tumor site		
Oral cavity	17	42.5
Upper gingiva	5	12.5
Tongue	4	10.0
Lower gingiva	3	7.5
Floor of mouth	2	5.0
Buccal mucosa	2	5.0
Hard palate	1	2.5
Oropharynx	7	17.5
p16-positive	3	7.5
p16-negative	4	10.0
Nasopharynx	5	12.5
Hypopharynx	3	7.5
Larynx	3	7.5
Paranasal sinus	3	7.5
External auditory canal	2	5.0
Target lesion (with duplicates)		
Oropharynx	24	
Anterior wall	8	
Lateral wall	7	
Posterior wall	5	
Superior wall	4	
Oral cavity	16	
Tongue	5	
Buccal mucosa	3	
Lower gingiva	3	
Hard palate	2	
Upper gingiva	2	
Floor of mouth	1	
Nasopharynx	6	
Cervical lymph node	5	
Maxillary sinus	5	
Nasal cavity	2	
External auditory canal	2	
Histology		
Squamous cell carcinoma	38	95.0
Adenoid cystic carcinoma	1	2.5
Lymphoepithelial carcinoma	1	2.5
T category		
0	4	10.0
1	12	30.0
2	8	20.0
3	8	20.0
4	8	20.0
N category		
0	35	87.5
1	1	2.5
2	0	0.0
3	4	10.0
M category		
0	39	97.5
1	1	2.5
History of surgery		
Yes	31	77.5
No	9	22.5
History of neck dissection		
Yes	28	70.0
No	12	30.0
History of radiotherapy		
Yes	38	95.0
No	2	5.0
History of systemic therapy		
Yes	8	20.0
No	32	80.0

ECOG, Eastern Cooperative Oncology Group.

**Table 2:** Best overall response.

Clinical Outcomes	Number	%
Objective response rate	30	75.0
Disease control rate	38	95.0
Complete response	11	27.5
Partial response	19	47.5
Stable disease	8	20.0
Progressive disease	2	5.0

**Table 3:** Adverse events.

Adverse Events	Patients, *n* (%)					
	Grade	Grade	Grade	Grade	Grade	All
	I	II	III	IV	V	
Pain	7 (17.5)	25 (62.5)	5 (12.5)	0 (0.0)	0 (0.0)	37 (92.5)
Mucositis	14 (35.0)	15 (37.5)	3 (7.5)	0 (0.0)	0 (0.0)	32 (80.0)
Hemorrhage	24 (60.0)	7 (17.5)	0 (0.0)	0 (0.0)	0 (0.0)	31 (77.5)
Dysphagia	8 (20.0)	5 (12.5)	6 (15.0)	0 (0.0)	0 (0.0)	19 (47.5)
Laryngeal edema	1 (2.5)	12 (30.0)	0 (0.0)	4 (10.0)	0 (0.0)	17 (42.5)
Edema of the face	10 (25.0)	3 (7.5)	5 (12.5)	0 (0.0)	0 (0.0)	18 (45.0)
Fistula	5 (12.5)	4 (10.0)	2 (5.0)	0 (0.0)	0 (0.0)	11 (27.5)
Sepsis	0 (0.0)	0 (0.0)	0 (0.0)	1 (2.5)	1 (2.5)	2 (5.0)
Infusion reaction	1 (2.5)	1 (2.5)	0 (0.0)	0 (0.0)	0 (0.0)	2 (5.0)
Facial nerve disorder	0 (0.0)	0 (0.0)	1 (2.5)	0 (0.0)	0 (0.0)	1 (2.5)
Lemierre’s syndrome	0 (0.0)	0 (0.0)	1 (2.5)	0 (0.0)	0 (0.0)	1 (2.5)
Pneumonitis	0 (0.0)	0 (0.0)	1 (2.5)	0 (0.0)	0 (0.0)	1 (2.5)
Liver dysfunction	0 (0.0)	0 (0.0)	1 (2.5)	0 (0.0)	0 (0.0)	1 (2.5)
Anemia	0 (0.0)	0 (0.0)	1 (2.5)	0 (0.0)	0 (0.0)	1 (2.5)
Acneiform rash	0 (0.0)	1 (2.5)	0 (0.0)	0 (0.0)	0 (0.0)	1 (2.5)
Vertigo	0 (0.0)	1 (2.5)	0 (0.0)	0 (0.0)	0 (0.0)	1 (2.5)
Fever	0 (0.0)	1 (2.5)	0 (0.0)	0 (0.0)	0 (0.0)	1 (2.5)
Retained needle fragment	0 (0.0)	1 (2.5)	0 (0.0)	0 (0.0)	0 (0.0)	1 (2.5)
Photosensitivity	1 (2.5)	0 (0.0)	0 (0.0)	0 (0.0)	0 (0.0)	1 (2.5)
Nausea	1 (2.5)	0 (0.0)	0 (0.0)	0 (0.0)	0 (0.0)	1 (2.5)

## Data Availability

Data are contained within the article.

## Abbreviations

The following abbreviations are used in this manuscript:

AE	Adverse event
BOR	Best overall response
CI	Confidence interval
CR	Complete response
DCR	Disease control rate
EGFR	Epidermal growth factor receptor
HN-PIT	Photoimmunotherapy for head and neck cancer
ICI	Immune checkpoint inhibitor
LA/LR-HNC	Locally advanced or locally recurrent head and neck cancer
ORR	Objective response rate
OS	Overall survival
PD	Progressive disease
PD-1	Programmed cell death protein 1
PFS	Progression-free survival
PIT	Photoimmunotherapy
PR	Partial response
QOL	Quality of life
RECIST	Response Evaluation Criteria In Solid Tumors
R/M HNSCC	Recurrent or metastatic head and neck squamous cell carcinoma
RWD	Real-world data
SD	Stable disease
TTF	Time to treatment failure

## References

[1] Ferlay J., Colombet M., Soerjomataram I., Mathers C., Parkin D.M., Piñeros M., Znaor A., Bray F. (2019). Estimating the global cancer incidence and mortality in 2018: GLOBOCAN sources and methods. Int. J. Cancer.

[2] Pulte D., Brenner H. (2010). Changes in survival in head and neck cancers in the late 20th and early 21st century: A period analysis. Oncologist.

[3] Wiegand S., Zimmermann A., Wilhelm T., Werner J.A. (2015). Survival after distant metastasis in head and neck cancer. Anticancer Res..

[4] Mitsunaga M., Ogawa M., Kosaka N., Rosenblum L.T., Choyke P.L., Kobayashi H. (2011). Cancer cell-selective in vivo near infrared photoimmunotherapy targeting specific membrane molecules. Nat. Med..

[5] Sato K., Ando K., Okuyama S., Moriguchi S., Ogura T., Totoki S., Hanaoka H., Nagaya T., Kokawa R., Takakura H. (2018). Photoinduced ligand release from a silicon phthalocyanine dye conjugated with monoclonal antibodies: A mechanism of cancer cell cytotoxicity after near-infrared photoimmunotherapy. ACS Cent. Sci..

[6] Hsu M.A., Okamura S.M., De Magalhaes Filho C.D., Bergeron D.M., Rodriguez A., West M., Yadav D., Heim R., Fong J.J., Garcia-Guzman M. (2023). Cancer-targeted photoimmunotherapy induces antitumor immunity and can be augmented by anti-PD-1 therapy for durable anticancer responses in an immunologically active murine tumor model. Cancer Immunol. Immunother..

[7] Nagaya T., Friedman J., Maruoka Y., Ogata F., Okuyama S., Clavijo P.E., Choyke P.L., Allen C., Kobayashi H. (2019). Host immunity following near-infrared photoimmunotherapy is enhanced with PD-1 checkpoint blockade to eradicate established antigenic tumors. Cancer Immunol. Res..

[8] Nakajima K., Takakura H., Shimizu Y., Ogawa M. (2018). Changes in plasma membrane damage inducing cell death after treatment with near-infrared photoimmunotherapy. Cancer Sci..

[9] Cognetti D.M., Johnson J.M., Curry J.M., Kochuparambil S.T., McDonald D., Mott F., Fidler M.J., Stenson K., Vasan N.R., Razaq M.A. (2021). Phase 1/2a, open-label, multicenter study of RM-1929 photoimmunotherapy in patients with locoregional, recurrent head and neck squamous cell carcinoma. Head Neck.

[10] Tahara M., Okano S., Enokida T., Ueda Y., Fujisawa T., Shinozaki T., Tomioka T., Okano W., Biel M.A., Ishida K. (2021). A phase I, single-center, open-label study of RM-1929 photoimmunotherapy in Japanese patients with recurrent head and neck squamous cell carcinoma. Int. J. Clin. Oncol..

[11] Okamoto I., Okada T., Tokashiki K., Tsukahara K. (2022). Quality-of-life evaluation of patients with unresectable locally advanced or locally recurrent head and neck carcinoma treated with head and neck photoimmunotherapy. Cancers.

[12] Nishikawa D., Suzuki H., Beppu S., Terada H., Sawabe M., Hanai N. (2022). Near-infrared photoimmunotherapy for oropharyngeal cancer. Cancers.

[13] Hirakawa H., Ikegami T., Kinjyo H., Hayashi Y., Agena S., Higa T., Kondo S., Toyama M., Maeda H., Suzuki M. (2024). Feasibility of near-infrared photoimmunotherapy combined with immune checkpoint inhibitor therapy in unresectable head and neck cancer. Anticancer Res..

[14] Nishikawa D., Shimabukuro T., Suzuki H., Beppu S., Terada H., Kobayashi Y., Hanai N. (2025). Predictive factors for the efficacy of head and neck photoimmunotherapy and optimization of treatment schedules. Cancer Diagn. Progn..

[15] von Elm E., Altman D.G., Egger M., Pocock S.J., Gøtzsche P.C., Vandenbroucke J.P. (2007). The Strengthening the Reporting of Observational Studies in Epidemiology (STROBE) statement: Guidelines for reporting observational studies. Lancet.

[16] Eisenhauer E.A., Therasse P., Bogaerts J., Schwartz L.H., Sargent D., Ford R., Dancey J., Arbuck S., Gwyther S., Mooney M. (2009). New response evaluation criteria in solid tumours: Revised RECIST guideline (version 1.1). Eur. J. Cancer.

[17] Brierley J.D., Gospodarowicz M.K., Wittekind C. (2017). TNM Classification of Malignant Tumours.

[18] National Cancer Institute (2017). Common Terminology Criteria for Adverse Events (CTCAE) Version 5. https://ctep.cancer.gov/protocolDevelopment/electronic_applications/ctc.htm#ctc_50.

[19] Kanda Y. (2013). Investigation of the freely available easy-to-use software “EZR” for medical statistics. Bone Marrow Transplant..

[20] Omura G., Honma Y., Matsumoto Y., Shinozaki T., Itoyama M., Eguchi K., Sakai T., Yokoyama K., Watanabe T., Ohara A. (2023). Transnasal photoimmunotherapy with cetuximab sarotalocan sodium: Outcomes on the local recurrence of nasopharyngeal squamous cell carcinoma. Auris Nasus Larynx.

[21] Koyama S., Ehara H., Donishi R., Taira K., Fukuhara T., Fujiwara K. (2024). Therapeutic host anticancer immune response through photoimmunotherapy for head and neck cancer may overcome resistance to immune checkpoint inhibitors. Case Rep. Oncol..

[22] Makino T., Sato Y., Uraguchi K., Naoi Y., Fukuda Y., Ando M. (2024). Near-infrared photoimmunotherapy for salivary duct carcinoma. Auris Nasus Larynx.

[23] Shibutani Y., Sato H., Suzuki S., Shinozaki T., Kamata H., Sugisaki K., Kawanobe A., Uozumi S., Kawasaki T., Hayashi R. (2023). A case series on pain accompanying photoimmunotherapy for head and neck cancer. Healthcare.

[24] Idogawa H., Shinozaki T., Okano W., Matsuura K., Hayashi R. (2023). Nasopharyngeal carcinoma treated with photoimmunotherapy. Cureus.

[25] Koyama S., Ehara H., Donishi R., Morisaki T., Ogura T., Taira K., Fukuhara T., Fujiwara K. (2023). Photoimmunotherapy with surgical navigation and computed tomography guidance for recurrent maxillary sinus carcinoma. Auris Nasus Larynx.

[26] Kishikawa T., Terada H., Sawabe M., Beppu S., Nishikawa D., Suzuki H., Hanai N. (2025). Utilization of ultrasound in photoimmunotherapy for head and neck cancer: A case report. J. Ultrasound.

[27] Suzuki T., Kano S., Suzuki M., Hamada S., Idogawa H., Tsushima N., Ashikaga Y., Wakabayashi Y., Soyama T., Hida Y. (2024). SlicerPIT: Software development and implementation for planning and image-guided therapy in photoimmunotherapy. Int. J. Clin. Oncol..

[28] Okada R., Ito T., Kawabe H., Tsutsumi T., Asakage T. (2024). Mixed reality-supported near-infrared photoimmunotherapy for oropharyngeal cancer: A case report. Ann Med. Surg..

[29] National Comprehensive Cancer Network (NCCN) Clinical Practice Guidelines in Oncology, Head and Neck Cancers Version 2.2025. https://www.nccn.org/professionals/physician_gls/pdf/head-and-neck_blocks.pdf.

[30] Ferris R.L., Blumenschein G., Fayette J., Guigay J., Colevas A.D., Licitra L., Harrington K., Kasper S., Vokes E.E., Even C. (2016). Nivolumab for recurrent squamous-cell carcinoma of the head and neck. N. Engl. J. Med..

[31] Burtness B., Harrington K.J., Greil R., Soulières D., Tahara M., de Castro G., Psyrri A., Basté N., Neupane P., Bratland Å. (2019). Pembrolizumab alone or with chemotherapy versus cetuximab with chemotherapy for recurrent or metastatic squamous cell carcinoma of the head and neck (KEYNOTE-048): A randomised, open-label, phase 3 study. Lancet.

[32] Okamoto I. (2025). Photoimmunotherapy for head and neck cancer: A systematic review. Auris Nasus Larynx.

[33] Shinozaki T., Matsuura K., Okano W., Tomioka T., Nishiya Y., Machida M., Hayashi R. (2023). Eligibility for photoimmunotherapy in patients with unresectable advanced or recurrent head and neck cancer and changes before and after systemic therapy. Cancers.

